# Prevalence, Causes, and Adverse Clinical Impact of Delayed Presentation of Non-COVID-19-Related Emergencies during the COVID-19 Pandemic: Findings from a Multicenter Observational Study

**DOI:** 10.3390/ijerph19169818

**Published:** 2022-08-09

**Authors:** Mohammed S. Alshahrani, Dunya Alfaraj, Jehan AlHumaid, Khalid Alshahrani, Aisha Alsubaie, Nasser Almulhim, Dana Althawadi, Salah Alam, Malak Alzahrani, Hassan Alwosibai, Abdullah Alshahrani, Rawan Makhdom, Faisal Alkhadra, Sukayna Al-faraj, Saad Al-Qahtani, Amal AlSulaibikh, Mohammed Al Jumaan, Laila Perlas Asonto, Sarah Alahmadi, Mohannad Alghamdi, Mohammed Al-Mulhim

**Affiliations:** 1 Emergency Medicine and Intensive Care Departments, King Fahd Hospital of the University, Imam Abdulrahman Bin Faisal University, Dammam 31441, Saudi Arabia; 2 College of Medicine, Imam Abdulrahman Bin Faisal University, Dammam 31441, Saudi Arabia; 3 Department of Emergency Medicine, King Fahd Hospital of the University, Imam Abdulrahman Bin Faisal University, Dammam 31441, Saudi Arabia; 4 Preventive Dental Sciences Department, College of Dentistry, Imam Abdulrahman Bin Faisal University, Dammam 31441, Saudi Arabia; 5 Department of Emergency, King Hamad University Hospital, Road 2835, Busaiteen P.O. Box 24343, Bahrain; 6 Emergency Department, King Fahad Hospital (KFU), Hofuf 36277, Saudi Arabia; 7 Department of Orthopedic Surgery, King Fahd Hospital of the University, Imam Abdulrahman Bin Faisal University, Dammam 31441, Saudi Arabia

**Keywords:** delayed presentation, non-COVID-19 emergencies, emergency department

## Abstract

Objective: The coronavirus disease (COVID-19) pandemic has disrupted healthcare systems worldwide, resulting in decreased and delayed hospital visits of patients with non-COVID-19-related acute emergencies. We evaluated the impact of the COVID-19 pandemic on the presentation and outcomes of patients with non-COVID-19-related medical and surgical emergencies. Method: All non-COVID-19-related patients hospitalized through emergency departments in three tertiary care hospitals in Saudi Arabia and Bahrain in June and July 2020 were enrolled and categorized into delayed and non-delayed groups (presentation ≥/=24 or <24 h after onset of symptom). Primary outcome was the prevalence and cause of delayed presentation; secondary outcomes included comparative 28-day clinical outcomes (i.e., 28-day mortality, intensive care unit (ICU) admission, invasive mechanical ventilation, and acute surgical interventions). Mean, median, and IQR were used to calculate the primary outcomes and inferential statistics including chi-square/Fisher exact test, t-test where appropriate were used for comparisons. Stepwise multivariate regression analysis was performed to identify the factors associated with delay in seeking medical attention. Results: In total, 24,129 patients visited emergency departments during the study period, compared to 48,734 patients in the year 2019. Of the 256 hospitalized patients with non-COVID-19-related diagnoses, 134 (52%) had delayed presentation. Fear of COVID-19 and curfew-related restrictions represented 46 (34%) and 25 (19%) of the reasons for delay. The 28-day mortality rates were significantly higher among delayed patients vs. non-delayed patients (*n* = 14, 10.4% vs. *n* = 3, 2.5%, OR: 4.628 (CI: 1.296–16.520), *p* = 0.038). Conclusion: More than half of hospitalized patients with non-COVID-19-related diagnoses had delayed presentation to the ED where mortality was found to be significantly higher in this group. Fear of COVID-19 and curfew restrictions were the main reasons for delaying hospital visit.

## 1. Introduction

An outbreak of atypical pneumonia caused by the spread of a novel coronavirus (severe acute respiratory syndrome coronavirus 2 (SARS-CoV-2)) in Wuhan, China was reported in December 2019. Consequently, SARS-CoV-2, with its high person-to-person transmissibility, subsequently spread to nearly all countries worldwide, with less lethality than the 2002–2003 SARS-CoV-1 [1]. Approximately 15% of patients with the coronavirus disease (COVID-19) exhibit severe symptoms and require hospitalization [2]. A proportion of those who enter the hospital setting require treatment in the intensive care unit (ICU), including advanced interventions for oxygenation. Worldwide, more than 248 million COVID-19 cases and more than 5 million COVID-19-related deaths have been reported from 223 countries as of September 2021 [3].

The COVID-19 pandemic has affected several aspects of the community and healthcare systems, predominantly due to the increased number of hospital visits by patients with symptoms of upper respiratory tract infection suspected to have COVID-19. Fear of contact with patients with COVID-19 and the government-imposed curfew might have resulted in reduced or delayed emergency department visits for other clinical illnesses. There is no consensus on the definition of delayed hospital presentation, which varies based on the symptoms and pathology.

Earlier studies have used multiple definitions for delayed hospital presentation. In this regard, the interval between symptoms’ onset and treatment initiation time has been considered most frequently [4]. Several contributory factors have been reported in the literature for delayed hospital presentation, including the pre-hospital phase, demographic characteristics, socioeconomic status, and ethnicity have been reported frequently as related factors for delayed hospital presentation [5,6,7]. Nonetheless, less studied factors, such as the type of clinical pathology, could play a role. Healthcare-system-related factors, such as structure, resources, and accessibility, are potentially important factors that need to be considered regarding delayed hospital presentation [8].

Few reports from different countries have described the impact of the delayed presentation of non-COVID-19-related conditions. In Hong Kong, a study that involved patients with ST-elevation myocardial ischemia (STEMI) revealed an apparent delay from the onset of chest pain to the first medical contact [9]. In a small case series (*n* = 12) from Italy, Lazzerini et al. found that half of the children in the series were admitted to an ICU, and four died; in all of the cases, the parents reported delayed hospital presentation because of the fear of SARS-CoV-2 infection [10]. Additionally, a study conducted by Clodfelder et al. concluded that 254 (8.12%) patients refused emergency services transportation before the pandemic compared to 479 (18.35%) during the pandemic (*p*-value < 0.001, chi square test) [11].

Several clinical conditions, such as sepsis, STEMI, and acute ischemic stroke, as well as surgical emergencies require time-sensitive treatments and interventions, and any delay in seeking medical attention might lead to worsening of the clinical condition and com-plications.

During the COVID-19 pandemic, attention has been focused mainly to diagnose and treat patients with COVID-19 while protecting others from the infection. Thus, the management of non-COVID-19-related illnesses received less attention. This study aimed to study the impact of the COVID-19 pandemic on the hospital presentation of patients with non-COVID-19-related illnesses and to determine the numbers, causes, and clinical outcomes of the patients with delayed hospital presentation.

## 2. Methods

### 2.1. Study Design and Setting

This is a multicenter, prospective, observational study conducted in two countries (Saudi Arabia and Bahrain) included all patients with non-COVID-19-related diagnoses who were hospitalized during the pandemic peak in June and July 2020 in three tertiary care centers: King Fahad Hospital of the University (KFHU), Dammam, Saudi Arabia; King Fahad Hospital (KFU), Hofuf, Saudi Arabia; and King Hamad University Hospital (KHUH), Bahrain. These hospitals are considered referral centers for suspected COVID-19 patients with availability of all healthcare subspecialties 24/7 with a capacity of more than 600 beds in each. They serve a total population of five million. The critical care unit’s capacity reached more than 50 patients with capabilities of extending the capacity by 50% in each hospital. The primary care center worked during the pandemic screening and treating low acuity cases. While high acuity cases directly approached emergencies in tertiary centers. The study followed the ethical standards of the institutional and national research committees and the Helsinki Declaration. Ethical approval for this study was obtained from the institutional review board at each participating center. This study followed the Strengthening the Reporting of Observational Studies in Epidemiology (STROBE).

### 2.2. Selection of Participants

Patients who presented to any of the emergency department at study centers with a non-COVID-19-related diagnosis and were hospitalized were included in this study. Based on the duration from symptom onset to emergency department presentation, the patients were assigned to either the delayed group (≥24 h) or the non-delayed group (<24 h). In the absence of a standard definition, the investigators chose the 24 h cutoff point to maximize the likelihood for a true definition of a delayed presentation. The inclusion criteria of this study were as follows: (1) adult patients (>18 years old) admitted from the emergency department; (2) non-COVID-19-related diagnosis; (3) acute illness with no pre-planned admission orders; and (4) negative results on the SARS-CoV-2 real-time polymerase chain reaction test. Patients who underwent elective admission, those who had terminal illnesses and were admitted for palliative care, and those who could not provide an accurate history of the timing of symptom onset or other important study-related data were excluded from this study. The eligible patients were contacted by the research team and voluntarily provided written informed consent for study participation. All prospective study participants were interviewed by one of the co-investigators at the time of admission or within 24 h of hospitalization. Patients were asked about the exact timing of symptom onset and their course of action thereafter. We collected information on demographics and socioeconomic condition, including sex, age, race, marital status, level of education, functional status, and mobility. For the patients in the delayed group, an interviewer-administered questionnaire was used. It included questions about the reasons for the delay and specifically enquired whether it was due to: (1) the fear of contact with patients with COVID-19; (2) advice by others or health authorities to delay the hospital visit; (3) restriction of movement due to curfew; or (4) other causes. The questionnaire was developed based on a literature review and a focus-group discussion with the investigators and was pilot-tested through 10 patients prior to study initiation.

### 2.3. Primary Outcome

The primary outcome of this study was to establish the prevalence of hospitalized patients with a non-COVID-19-related emergency diagnosis who had evidence of delayed hospital presentation.

### 2.4. Secondary Outcome

The secondary outcomes included identification of the causes of the delayed presentation as: (1) fear of contact with patients with COVID-19; (2) advice by others or health authorities to delay the hospital visit; (3) restriction of movement due to curfew; (4) clinical outcomes in terms of 28-day mortality; need for ICU/cardiac care unit (CCU) admission; need for mechanical ventilation (invasive/noninvasive); need for vasopressor therapy; need for emergency surgery (with 24 h of presentation); and length of hospital stay; and (5) other causes.

### 2.5. Statistical Analyses

Descriptive data are presented as frequencies (percentages), for discrete variables, and means (standard deviation (SD)) or medians (interquartile range (IQR)) for continuous variables. To examine the differences between groups of patients, Student’s t test was used for continuous variables as appropriate, and Chi-square test or Fisher’s exact test was used for differences in categorical variables as appropriate. To determine whether the time of treatment, either from the onset of symptoms or from hospital or intensive care unit admission have an impact on survival, we performed a Cox-proportional hazards model with time of treatment as a time-dependent covariate and simultaneously adjusting for all other confounders. Stepwise regression analysis was performed, variables with the significance level less than 0.10 in univariate analysis were considered as potential cofounders. Then, using only significant cofounders, a multivariate regression model was built. The Kaplan–Meier curves were used to determine the probability of survival among the delayed and no delayed patient over the length of stay in hospital. A log-rank test was used to compare these curves. All statistical tests were two-tailed. Factors were considered statistically significant at a *p* < 0.05. Additionally, the study conducted univariate and multiple regression analyses of factors (patient characteristics) that were potentially associated with 28-day mortality rate in the delayed group which included age, sex, ethnicity, level of education, and organ system involved.

## 3. Results

In total, 24,129 patients visited the emergency departments at the three study centers during the study period (June–July 2020), compared with 48,734 patients during the same period in year 2019, which indicated a 50.49% decrease in the inflow of patients during the COVID-19 crisis. Based on the pre-specified study eligibility criteria, 256 patients were included in the study. In total, 109 (43%) were from the KFHU, 69 (27%) were from KFH, and 78 (31%) were from KHUH. More than half of the participants (134/256; 52%) were included in the delayed group. The delayed and non-delayed groups did not differ significantly in the proportions of male and female participants (55% and 45% vs. 56% and 45% (*p* = 0.517)). The mean age for participants in the delayed and non-delayed groups was 51.8 and 50.9 years (*p* = 0.601). There were no significant between-group differences in the proportions of participants living alone and in their level of education, marital status, and mobility (Table 1).

Table 2 presents comparisons of vital signs and important basic laboratory findings on presentation to the emergency department. The patients in the delayed group had a lower hemoglobin level than the patients in the non-delayed group (11.4 ± 2.8 vs. 12.1 ± 2.6 g/dL; *p =* 0.043). However, there was no significant intergroup difference in the heart rate, oxygen saturation, respiratory rate, and the other important laboratory findings. In both groups, the blood pressure and temperature were within normal ranges at presentation in the emergency department.

The mortality rate was significantly higher in the delayed group than in the non-delayed group (*n* = 14 (10.4%) vs. 3 (2.5%), OR: 4.628 (CI: 1.296–16.520), *p* = 0.038). The need for ICU/CCU admissions and invasive mechanical ventilation was not significantly higher in the delayed group than in the non-delayed group (*n* = 38 (28.4%) vs. 22 (18%), OR: 1.799 (CI: 0.992–3.262), *p* = 0.051 and 17 (12.7%) vs. 9 (7.4%), OR: 1.824 (CI: 0.781–4.261), *p =* 0.160, respectively). There was no significant difference in the proportion of patients who needed acute surgical intervention within 72 h of admission between the delayed and non-delayed groups (17 (12.7%) vs. 9 (7.4%), OR: 1.824 (CI: 0.781–4.261), *p* = 0.16). The length of hospital stay was shorter in the non-delayed group (mean = 4 (interquartile range: 2–7 days); Table 3). Risk was higher among the delayed presented patients for all severe indicators (died, ICU needed, emergency surgery required, and vasopressor needed; Table 3).

**Figure 1:**
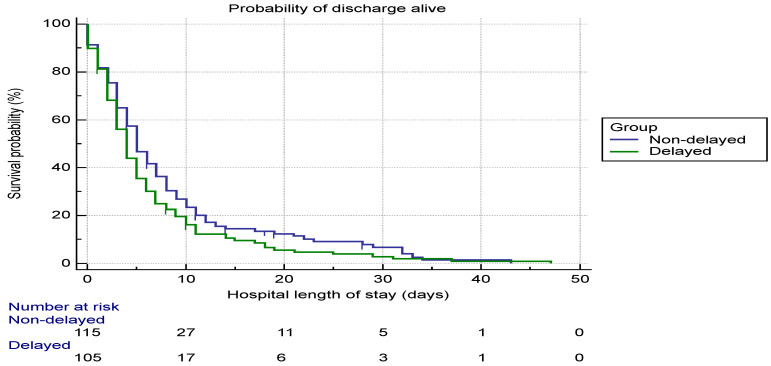
Kaplan–Meier curve of the probability of being alive at discharge among delayed and non-delayed patients based on the length of hospital stay.

The Kaplan–Meier curve showed a higher probability of survival in the non-delayed group than in the delayed group (Figure 1). Patients who showed up early (non-delayed) were more likely to discharge alive (*p* = 0.001).

The main cause of delayed emergency department presentation and 28-day outcome are described in Table 4. Among the causes of delayed presentation, the fear of SARS-CoV-2 infection ranked highest (*n* = 46; 34%), followed by the restriction of movement due to curfew (*n* = 25; 19%) and advice from others (*n* = 20; 15%).

Table 5 lists the possible factors that were hypothesized to be associated with the delay in seeking medical treatment its relation to 28-day mortality rate. Univariate Cox analysis showed that, in the delayed group, lower age was significantly associated with longer survival (hazard ratio (HR): 0.99, 95% CI (0.980–1.00), whereas a higher education level was positively associated with a high probability of survival HR: 2.201, 95% CI (1.25–3.85); invasive mechanical ventilation at the time of admission was negatively associated with survival HR: 0.139, 95% CI (0.030–0.641); direct admission to the ICU/CCU was associated with a greater probability of survival HR: 1.48, 95% CI (0.199–11.04). In the joint impact-of-survival analysis, only the need for mechanical ventilation persisted as a significant factor (*p* < 0.05).

## 4. Discussion

The results from this multicenter observational showed a significant reduction in emergency department visits in the study centers during the pandemic peak. This study cohort of non-COVID-19-related patients showed significant delay (>24 h) in hospital presentation. In 53% of the delayed group, the main reason was either the fear of contact with patients with COVID-19 or the restriction of movement due to curfew. The delay had a negative impact on the clinical outcome of patients in the delayed group in terms of mortality compared with the non-delayed group.

This study highlights the magnitude of the collateral damage caused by the COVID-19 pandemic to the healthcare system, and the impacts are evident worldwide about the reduction in hospital visits of patients with non-COVID-19-related illnesses and the intentional delay of their presentation, due to either fear of contracting COVID-19 or the restriction of movement imposed by the governments in many countries. Concomitantly, there are anecdotal reports from different parts of the world that carry the same concerns. In Italy, a small case-series that included 12 children with non-COVID-19-related illness reported a significant delay in hospital presentation even though half of them were admitted to the ICU, and four died. All families reported avoiding hospital visits due to fear of COVID-19 infection; the cases included medical and surgical emergencies with delays of up to a few days [10]. In the same report in Italy, a reduction in emergency department visits was likewise observed. A survey of more than 2000 pediatricians in the UK found that 32% had witnessed delayed presentations where cases included newly diagnoses diabetes mellitus with diabetic ketoacidosis, sepsis, and malignancy [12].

The same concern was raised by physicians practicing in New York City at the peak of the pandemic, where they encountered patients with delayed presentations in many acute, non-COVID-19-related diseases, such as delayed presentation in acute coronary syndrome despite typical symptoms that lasted for a few days and progressed quickly to cardiogenic shock and death. In the same report, breast oncologists reported that many patients easily accepted the treatment modifications from surgical to hormonal therapy to avoid hospitals despite the uncertainty about the efficacy of the alternative option. The chemotherapy sessions and surgical oncological procedures of some cancer patients were delayed due to the fear of office visits in such immunocompromised patients. The report from New York City highlighted the negative impact of the pandemic on surgical practices where elective surgical procedures that were postponed included oncologic, cardiac, and other elective surgical procedures, and some patients died while awaiting treatment [13]. Some other acute life-threatening surgical emergencies have been reported during the pandemic for the same reasons; for example, a case report of a large necrotic abdominal abscess with a concern of necrotizing fasciitis was left untreated for 3 weeks and needed emergency surgery and wound exploration and debridement [13,14].

Another case series from New Jersey reported 10 cases of myocardial infarction with late presentation and identified unusual post-MI complications, such as ventricular septal rupture, left ventricular pseudo aneurysm, and right ventricular infarction. The main reason for not seeking medical advice was the fear COVID-19 exposure within healthcare facilities [15]. Similar findings were reported for the neurological emergency of ischemic stroke; a study of 710 consecutive patients with acute ischemic strokes who presented to 12 stroke centers across the US during the COVID-19 pandemic found a significant delay in presentation in the study group [16]. This population-based cross-sectional study reported a 400% increase in at-home cardiac arrests in New York City during the peak of the pandemic compared with the same period in the previous year. Older patients, non-white ethnicity, hypertension, diabetes, and physical limitations were found to be more prevalent in that cohort [17].

Similar collateral negative effects were encountered previously during the SARS outbreak in early 2003, and during the peak of that epidemic, Taiwan saw a significant reduction in patients seeking ambulatory, inpatient, and dental care. A main reason for this reduction was that the fear of SARS-CoV-1 infection influenced people’s willingness and choice to seek appropriate medical care [6]. These concerns were reflected in a study that included patients with cancer at the Taipei Veterans General Hospital, Taiwan, as 63.8% were scared to visit hospitals during the SARS epidemic and 36.2% felt that SARS was more severe and more fatal than their underlying cancer [7]. As compared to our study, 34% of our patients included in the study had a delayed presentation because of fear of COVID-19 exposure while 19% was because of the curfew restrictions.

In 2022, Anderson et al. concluded that they did not observe any reduction in ED visits nor an increase in the presentation of sicker patients with urinary tract stones during the pandemic. However, this article was conducted in one center, and it included a relatively small number of patients. Additionally, their selection was based on patients who had positive findings in CT KUB scans, excluding patients diagnosed based on other modalities or patients who did not undergo further radiological procedures. Lastly, they compared the patients’ ED visits of urinary tract stones 100 days before and during the pandemic. At that time, there was a vacation period in the pre-pandemic period, and the ED visits were usually lower at that time of the year compared to the next period. On the other hand, our study is multi-center, with a larger patient number and was compared to the ED visits for the same period one year before the pandemic [18].

We believe that this study has multiple strengths in its prospective, multicenter design and being conducted in two different countries as well as the enrolment of consecutive non-COVID-19 patients. Moreover, all related important clinical outcomes were reported, and all enrolled participants were followed up for 28 days. Further studies should be conducted with a larger sample size for a better evaluation of the pandemic’s effects on non-COVID-19-related presentations.

## 5. Limitations

The study has some limitations in that the observational nature of the study may not prevent the inclusion of confounding factors that led to the negative clinical outcome in the delayed group. Furthermore, only about half of the delayed group clearly stated that the cause of their delay was associated with the pandemic, either due to the fear of COVID-19 infection or because of curfew-related reasons. There is a possibility that some patients hide the main reason for the delay to avoid being blamed. In addition, this study was designed to exclude the cases discharged from ED by a medical decision where this decision might contribute into a delayed presentation. Larger studies might be needed to confirm this observation.

## 6. Conclusions

In summary, this study showed that the impact of the COVID-19 pandemic is not limited to SARS-CoV-2 infection and its complications. Patients with non-COVID-19-related disease had significantly delayed presentations, which led to negative clinical outcomes in a considerable number of patients. This information might be useful in planning better access to healthcare in future pandemic outbreaks to ensure that care should not be limited to patients who have been infected and essential attention must likewise be geared toward the non-pandemic-related illnesses to prevent poor clinical outcomes.

## Figures and Tables

**Table 1:** Demographics and baseline characteristics of patients presenting to the hospital.

Demographic Variables		Delayed (*n* = 134), *n* (%)	Non-Delayed (*n* = 122), *n* (%)	*p*-Value
Age, years		51.78 ± 18.3	50.88 ± 19.8	0.601
Sex	Male	74 (55)	68 (56)	0.517
	Female	60 (45)	54 (45)	
Marital status	Married	93 (69)	82 (67)	0.404
	Single	41 (31)	40 (32)	
Living status	Alone	14 (10)	13 (11)	0.558
	With family	120 (90)	109 (89)	
Education level	Primary	35 (26)	20 (16)	0.291
	Secondary	52 (39)	47 (39)	
	College	29 (22)	31 (26)	
	Postgraduate	4 (3)	7 (6)	
	Illiterate	14 (10)	17 (14)	
Mobility level	Dependent	35 (26)	27 (22)	0.275
	Independent	99 (74)	95 (78)	
Ethnicity	Arab	115 (86)	101 (83)	0.596
	Asian	9 (7)	6 (5)	
	South Asian	7 (5)	12 (10)	
	Other Black	1 (1)	2 (2)	
	European	2 (2)	1 (1)	

**Table 2:** Comparisons of vital signs and laboratory test results at the time of admission among the patients who presented to the emergency department.

Vitals/Laboratory Tests	Delayed	Non-Delayed	*p*-Value
Heart rate, beats/min	96.9 ± 18.3	93.9 ± 19.5	0.179
Blood pressure, systolic/diastolic, mmHg	134.3 ± 32.4/78.4 ± 20.2	137.1 ± 26.9/78 ± 14.3	0.447/0.851
Respiratory rate, cycles/min	21.1 ± 4.2	20.6 ± 5.3	0.412
Temperature, °C	36.7 ± 3.2	36.9 ± 0.5	0.479
Oxygen saturation, %	96.8 ± 4.6	97.7 ± 5.1	0.123
Hemoglobin, g/dL	11.4 ± 2.8	12.1 ± 2.6	0.043 *
White blood cell count, per µL	11.7 ± 5.6	11.7 ± 16.6	0.992
Platelet count	286 ± 120.1	287.7 ± 106.9	0.903
Glucose, mg/dL (Median, IQR) ^∞^	142 (90–200)	117 (45–178)	0.149
Creatinine, mg/dL (Median, IQR) ^∞^	1.10 (0.8–1.7)	1.10 (0.8–1.73)	0.680

Data are presented as mean ± SD and ^∞^ Median (IQR). * *p* < 0.05.

**Table 3:** Outcomes of delayed and non-delayed patients.

Characteristics	Delayed *n* = 134 *n* (%)	Non-Delayed *n* = 122 *n* (%)	Odd Ratio (95% CI)	Risk Ratios	*p*-Value
Outcome					
Died	14 (10.4)	3 (2.5)	4.628 (1.296 to 16.520)	3.38	0.038 *
Admission type					
ICU	38 (28.4)	22 (18.0)	1.799 (0.992 to 3.262)	1.57	0.051
Non-ICU	96 (71.6)	100 (82.0)			
Respiratory data on admission					
Invasive mechanical ventilation	17 (12.7)	9 (7.4)	1.824 (0.781 to 4.261)	4.61	0.024 *
Noninvasive ventilation	117 (87.3)	113 (92.6)			
Emergency surgery					
Yes	17 (12.7)	9 (7.4)	1.824 (0.781 to 4.261)	1.248	0.160
No	117 (87.3)	113 (92.6)			
Vasopressor therapy					
Yes	10 (7.5)	3 (2.5)	3.199 (0.859 to 11.909)	3.03	0.069
No	124 (92.5)	119 (97.5)			
Length of hospital stay, days **	5 (2–9)	4 (2–7)			0.082

* *p* < 0.05. Data are presented as ** median (interquartile range), percentages and odds ratio and relative risk ratio; ICU = intensive care unit.

**Table 4:** The 28-day outcome stratified according to the cause of delayed presentation.

Outcome	Causes of Delayed Presentation			
	Fear of COVID-19	Advice by Other Healthcare Providers	Movement Restriction Due to Curfew	Others
Died	4 (3)	2 (2)	5 (4)	0 (0)
Discharged	37 (28)	17 (13)	18 (13)	36 (27)
Hospitalized currently	3 (2)	0 (0)	2 (2)	1 (1)
Lost to follow-up	2 (2)	1 (1)	0 (0)	5 (3)
DAMA/absconded	0 (0)	0 (0)	0 (0)	1 (1)
Total	46 (34)	20 (15)	25 (19)	43 (31)

**Table 5:** Results of Cox univariate and multiple regression analyses of factors that are potentially associated with >24 h delay in presentation to the emergency department and 28-day mortality rate.

Characteristics	Possible Factors	Univariate Analysis		Multivariate Analysis	
		Hazard Ratio	95% CI	Hazard Ratio	95% CI
	Age	0.99 *	0.980–1.00	0.993	0.980–1.00
Education level	Primary	Ref			
	Secondary	1.711 *	1.056–2.77	1.247	0.762–2.13
	College	2.201 *	1.25–3.85	1.51	0.782–2.93
	PG	2.448	0.84–7.15	1.77	0.581–5.44
Invasive mechanical ventilation	No	Ref			
	Yes	0.139 *	0.030–0.641	0.104 **	0.021–0.532
ICU/CCU care needed at Admission	No	Ref			
	Yes	1.484	0.199–11.04	3.521	0.431–28.79
Vasopressor therapy	No	Ref			
	Yes	0.333 *	0.145–0.763	0.452	0.178–1.114

* *p* < 0.100, ** *p* < 0.05. The analyses were adjusted for sex, ethnicity, mode of transportation, mobility, basic lab test, and admission diagnosis. ICU = intensive care unit; CCU = cardiac care unit.

## Data Availability

All data produced and analyzed in this study are included in this manuscript as presented in Table 1, Table 2, Table 3, Table 4 and Table 5 and Figure 1. The manuscript does not contain any individual person’s data. Standing Committee for Research Ethics on Living Creatures (SCRELC) and the authors have no objection in granting and assigning this journal unrestricted right to reproduce, publish, and distribute this manuscript in all forms.
